# The time-dependent changes in serum immunoglobulin after kidney transplantation and its association with infection

**DOI:** 10.3389/fimmu.2024.1374535

**Published:** 2024-04-19

**Authors:** Eun-Ah Jo, Sangil Min, Ae jung Jo, Ahram Han, Jongwon Ha, Eun Young Song, Hajeong Lee, Yong Chul Kim

**Affiliations:** ^1^ Department of Surgery, Seoul National University College of Medicine, Seoul, Republic of Korea; ^2^ Department of Surgery, Chung-Ang University Hospital, Seoul, Republic of Korea; ^3^ Department of Information Statistics, Andong National University, Andong, Republic of Korea; ^4^ Department of Laboratory Medicine, Seoul National University College of Medicine, Seoul, Republic of Korea; ^5^ Department of Nephrology, Seoul National University College of Medicine, Seoul, Republic of Korea

**Keywords:** immunoglobulin, complement levels, kidney transplantation, immunosuppression, infection

## Abstract

**Introduction:**

Kidney transplant recipients often experience significant alterations in their immune system, which can lead to increased susceptibility to infections. This study aimed to analyze time-dependent changes in serum immunoglobulin and complement levels and determine the risk factors associated with infection.

**Methods:**

A retrospective analysis of serum samples from 192 kidney transplant recipients who received transplantations between August 2016 and December 2019 was conducted. The serum samples were obtained at preoperative baseline (T0), postoperative 2 weeks (T1), 3 months (T2), and 1 year (T3). The levels of serum C3, C4, IgG, IgA, and IgM were measured to evaluate immune status over time.

**Results:**

The analysis revealed significant decreases in IgG and IgA levels at T1. This period was associated with the highest occurrence of hypogammaglobulinemia (HGG) and hypocomplementemia (HCC), as well as an increased incidence of severe infection requiring hospitalization and graft-related viral infections. Using a time-dependent Cox proportional hazards model adjusted for time-varying confounders, HGG was significantly associated with an increased risk of infection requiring hospitalization (HR, 1.895; 95% CI: 1.871–1.920, P-value<0.001) and graft-related viral infection (HR, 1.152; 95% CI: 1.144–1.160, P-value<0.001).

**Discussion:**

The findings suggest that monitoring serum immunoglobulin levels post-transplant provides valuable insights into the degree of immunosuppression. Hypogammaglobulinemia during the early post-transplant period emerges as a critical risk factor for infection, indicating that serum immunoglobulins could serve as feasible biomarkers for assessing infection risk in kidney transplant recipients.

## 1 Introduction

The occurrence of infection after solid organ transplantation (SOT) results in increased morbidity and mortality (1–5). Consequently, many studies have attempted to identify risk factors and potential predictors of infection. Due to the suppression of cellular immunity through immunosuppressant usage, the monitoring of innate and humoral immunity by quantification of serum immunoglobulin and complement levels has been especially suggested as potential biomarkers of infection in transplant recipients (6, 7).

Hypogammaglobulinemia (HGG) has been extensively studied in SOT and has shown associations with bacterial, cytomegalovirus (CMV), and fungal infection (2, 3, 8). However, differences in the definitions for post-transplant infection and timing of immunoglobulin monitoring have resulted in discrepancies across studies (2). Contrary to heart or lung transplant recipients, renal transplant recipients showed a dampened decrease in IgG levels, and the relationship to infection showed contradictory results (1, 2, 9).

Similarly, serum C3 and C4 are good candidates for monitoring the complement system (9, 10). Each component of the complement system activates the complement cascades through intricate interactions between various soluble products and cell surface receptors and plays a crucial role against infection (8, 11). C4 has a central role in the classical pathway and has shown associations with early and late allograft function (8, 12). All three activation cascades—classical, alternative, and lectin pathway—converge at the level of C3 to form C5 convertase and result in the formation of the membrane attack complex (1, 10, 12). Decreased levels of C3 have been reported to be associated with the occurrence of infectious complications after heart and lung transplantation (7, 11, 13). However, the relationship between hypocomplementemia (HCC) and infection is yet controversial (7). Some studies have even reported an association between increased levels of C3 and infections such as CMV infection (4, 11, 12, 14).

These inconsistencies may be due to individual situations that result in varying degrees of dysregulation of immune function and susceptibility such as immunosuppressive medications, desensitization, rejection treatment, and infection itself (2). To reflect the different changes in immune function and severity of immunosuppression, this study aimed to analyze the time-dependent changes in serum immunoglobulin and complement levels of individual kidney transplant recipients and identify risk factors associated with infection.

## 2 Methods

### 2.1 Study design and data collection

A retrospective study using serum samples from a prospective biobank of kidney transplant recipients at Seoul National University Hospital from August 2016 to December 2019 was undertaken. Routine serum samples were collected preoperatively (T0) and at 2 weeks postoperatively (T1), 3 months postoperatively (T2), and 1 year postoperatively (T3). Data on clinical variables, transplantation admission and operation history, cause of end-stage renal disease, lab findings, immunosuppressive therapies, and rejection and infection history were extracted and categorized based on this time interval (T0–T3).

The occurrence of specific episodes, such as infection and rejection, was defined according to the intervals between the serum samples collected: P0 if it occurred between T0 and T1, P1 if it occurred between T1 and T2, P2 if it occurred between T2 and T3, and P3 if it occurred within 3 months after T3 sampling. Infections that occurred in P0 were excluded from the analysis as they overlapped with admission for transplantation and might not reflect true opportunistic infection.

During the study period, a total of 569 kidney transplantations were performed. Excluding patients with missing samples from any of the four time periods, a total of 192 patients had all four samples and were included for the final analysis.

The study was designed to evaluate (1) the serial changes in serum levels of immunoglobulins (IgG, IgA, and IgM) and complement components (C3 and C4) across four key postoperative intervals (T0, T1, T2, and T3) to assess the temporal changes in immune function, (2) the prevalence of hypogammaglobulinemia (HGG), hypocomplementemia (HCC), and infection, and (3) the association between HGG and infection to identify significant risk factors for infection among transplant recipients.

### 2.2 C3, C4, IgG, IgA, and IgM measurement

The serum samples from the biobank were preserved in liquid nitrogen tanks at -195°C and thawed before use. The IgG, IgA, IgM, C3, and C4 levels collected at each time period (T0–T3) were measured using an automated chemical analyzer (TBA-Fx8, Toshiba, Tokyo, Japan) and immunoassay reagents supplied by the manufacturer (Abott, Tokyo, Japan).

Based on the measured serum levels, hypogammaglobulinemia (HGG) was defined as a serum IgG level below the normal range or the presence of more than one class of immunoglobulins below the normal range. The normal ranges were defined as follows: IgG, 700–1,600 mg/dL; IgA, 70–400 mg/dL; and IgM, 40–230 mg/dL (15). Hypocomplementemia (HCC) was defined as serum C3 (hypoC3) or C4 (hypoC4) levels below the normal range. The normal ranges were defined as follows: C3, 83–171 mg/dL and C4, 14–38 mg/dL (11).

### 2.3 Infection definition

The infection episodes that were analyzed were severe infection, defined as any infectious episode requiring hospitalization and treatment, and graft-related viral infections, defined as infection by any of the following pathogens: polyoma BK virus (BKV), cytomegalovirus (CMV), and Epstein–Barr virus (EBV). CMV, EBV, and BKV are routinely screened at our center through serum quantitative polymerase chain reaction (qPCR) preoperatively and at 2 weeks postoperatively, 3 months postoperatively, 6 months postoperatively, and 1 year postoperatively. BKV infection was defined as viremia with a viral load over 1,000 copies/mL detected through qPCR or biopsy-proven BKV-associated nephropathy. EBV infection was defined as viremia with a viral load over 4,000 copies/mL detected through qPCR. CMV infection was defined as viremia with a viral load over 1,000 copies/mL detected through qPCR with or without signs and symptoms of disease or the presence of specific symptoms in a target organ with positive CMV in tissue culture.

Patients who underwent preoperative desensitization and rejection treatment received *Pneumocystis jirovecii* prophylaxis of 400 mg trimethoprim/80 mg sulfamethoxazole daily for 6 months. CMV prophylaxis for 6 months with valacyclovir was administered to patients who underwent induction treatment with anti-thymoglobulin or was a CMV-seronegative recipient with a CMV-seropositive donor (CMV D+/R-).

### 2.4 Immunosuppression

All patients were prescribed a triple immunosuppressant regimen comprised of tacrolimus, mycophenolate mofetil, and steroid. For the first 3 months post-transplantation, the tacrolimus trough level (FK C0) was targeted to 8–12 ng/mL. The target levels were then lowered to 6–10 ng/mL until 1 year after transplantation and to 4–6 ng/mL thereafter. Mycophenolate mofetil was administered at a fixed dose of 500 mg twice a day. IV steroids of 500 mg were used on the day of operation, followed by rapid tapering to achieve a daily dose of oral prednisolone at 5 mg within the first month after transplantation.

Incompatible living donor transplantation (iLDKT) was defined as recipients who received kidneys from ABO-incompatible donors or had pre-formed donor-specific antibodies (DSA). The desensitization protocol for such iLDKT recipients was as follows: rituximab administration 2–4 weeks prior to transplantation, followed with pretransplant plasmapheresis (PPH) with intravenous immunoglobulin (IVIG) of 0.2 g/kg prior to transplantation until the ABO antibody titer levels dropped below 1:32 and the DSA MFI levels below 1,000.

For induction immunosuppression, basiliximab was administered (20 mg) at days 0 and 4 after transplantation. In high-risk patients with high panel-reactive antibody titers (PRA) (>80%) or who were initially DSA-positive, anti-thymoglobulin (ATG) (1.5 mg/kg/day) was administered instead of basiliximab and was maintained until postoperative day 3 or 4.

### 2.5 Rejection

A rejection event was defined as any biopsy-proven rejection, including borderline rejection, defined in accordance with the Banff ‘17 classification by an experienced renal pathologist. Biopsy was undertaken as per protocol at reperfusion and at 2 weeks and 1 year postoperatively or performed when indicated due to worsening of the graft, defined as greater than 20% increase in baseline serum creatinine or an appearance of *de novo* DSA to human leukocyte antigen.

If clinically suspected, rejection treatment with steroid pulse therapy was undertaken immediately after biopsy without waiting for histologic confirmation. All other forms of rejection treatment were started after a histologic diagnosis was made by the pathologist.

### 2.6 Statistical analysis

Quantitative data were shown as mean ± standard deviation (SD), and qualitative data were presented as relative frequencies (%). The Wilcoxon signed-rank test with Bonferroni correction was used to evaluate the statistical significance of the temporal changes in serum IgG, IgA, IgM, C3, and C4 levels at the four different time points (T0–T3).

Given the potential of immune modulating covariates in transplant recipients to act as risk factors for hypogammaglobulinemia, we initially conducted logistic regression using generalized estimating equations (GEE). As the outcome variable HGG was measured repeatedly, GEE was used to adjust for the clustering effects within one patient. Variables known to impact and cause HGG were selected as variables for univariate analysis. Baseline factors such as age, BMI, dialysis state, iLDKT, underlying diseases (DM, pulmonary disease, and liver disease), CMV serology and treatment, immunosuppressant use (preoperative desensitization and induction and postoperative rejection and treatment), and low immune function such as baseline HGG or HCC were selected for analysis. Then, a stepwise backward variable selection method was performed using significant variables from the univariate analysis.

A time-dependent Cox proportional hazards model was then employed to explore the relationship between hypogammaglobulinemia and infection. To further account for time-varying exposure of HGG on the occurrence of severe infection and graft-related viral infection, we utilized a marginal structural Cox proportional hazards model with stabilized weights derived from inverse probability treatment weighting (IPTW) (16, 17). The marginal structural model statistically adjusted for the confounding effect of the imbalanced variables on both the probability that an individual will be allocated to two HGG states (having HGG or not having HGG) as well as the probability of the occurrence of infection at a certain time point. The stabilized weight at time *t* consisted of the product of the treatment weight and censoring weight and was as follows, where A(k) was the HGG state at time *k*, and $\bar{A}$ (k-1) was the HGG state prior to time *k*:


$$\text{s}{\text{w}}_{\text{i}}(\text{t})=\prod_{\text{k}=1}^{\text{t}}\frac{\text{pr}(\text{A}(\text{k})={\text{a}}_{\text{i}}(\text{k})|\bar{\text{A}}(\text{k}-1)=\bar{{\text{a}}_{\text{i}}}(\text{k}-1),\text{V}={\text{v}}_{\text{i}})}{\text{pr}(\text{A}(\text{k})={\text{a}}_{\text{i}}(\text{k})|\bar{\text{A}}(\text{k}-1)=\bar{{\text{a}}_{\text{i}}}(\text{k}-1),\bar{\text{L}}(\text{k})=\bar{{\text{l}}_{\text{i}}}(\text{k}),\text{V}={\text{v}}_{\text{i}}}$$

$$\text{s}{\text{w}}_{\text{i}}{\left(\text{t}\right)}^{\text{c}}=\prod_{\text{k}=1}^{\text{t}}\frac{\text{pr}(\text{C}(\text{k})=0|\bar{\text{C}}(\text{k}-1)=0,\bar{\text{A}}(\text{k}-1)=\bar{{\text{a}}_{\text{i}}}(\text{k}-1),\text{V}={\text{v}}_{\text{i}})}{\text{pr}(\text{C}(\text{k})=0|\bar{\text{C}}(\text{k}-1)=0,\bar{\text{A}}(\text{k}-1)=\bar{{\text{a}}_{\text{i}}}(\text{k}-1),\bar{\text{L}}(\text{k}-1)=\bar{{\text{l}}_{\text{i}}}(\text{k}-1),\text{V}={\text{v}}_{\text{i}})}$$

Similarly, C(k) was the censoring state at time *k*, $\bar{C}$ (*k* -1) was the history, $\bar{L}$ (*k*) (was the covariate history before time *k* and including time *k*, and *V* was the time-independent covariate observed on the index date.

Treatment weights were calculated based on the inverse of each individual’s probability of belonging to one of the two HGG states at each observed time point. Both time-dependent and time-independent covariates were considered in this calculation. Similarly, censoring weights were calculated based on the probability of being censored at each time point and considering both the time-dependent and time-independent covariates. The covariates that were included for calculating stabilized weights were immune modulatory factors. The time-dependent covariates were rejection event occurrence, rejection treatment with IV steroid, rejection treatment with plasmapheresis and immunoglobulin, rejection treatment with ATG, and rejection treatment with other antibodies. Time-independent covariates were preoperative desensitization including plasmapheresis, immunoglobulin usage and antibody treatment, and induction with anti-thymoglobulin. This approach aimed to provide an unbiased estimation of the causal effect of HGG levels on infection risk.

All *P*-values were two tailed, and a *P*-value of<0.05 was considered statistically significant. Statistical analysis was performed using SPSS version 27.0 (SPSS Inc., Chicago, IL, USA) and SAS 9.4 (SAS institute Inc., Cary, NC, USA) for Windows.

### 2.7 Ethics

The study was approved by the Institutional Review Board at Seoul National University Hospital (IRB no. 2106-094-1228). The need for informed consent was waived due to the retrospective nature of the study.

## 3 Results

### 3.1 Patient and transplantation characteristics

During the study period, a total of 569 kidney transplantations were performed. Among them, 192 patients had serum samples taken at all four time periods and were included in the study. None of these patients experienced graft failure or mortality within the study period. Most of the transplantations were living donor transplantations (81.8%, *n* = 157), of which 27.6% had incompatible donors. Preoperative desensitization was performed in a total of 50 patients (26%). Rituximab was administered to 48 patients (25%), and plasmapheresis with IVIG was performed in 32 patients (16.7%). Induction with ATG was undertaken in 25 patients (13%). Infection prophylaxis was undertaken for CMV in 35 patients (18.2%) and for *Pneumocystis pneumonia* (PCP) in 17 patients (8.9%). A total of 84 patients (43.8%) had at least one rejection event within the study period. Of these patients, 60 (71.4%) were pathologically borderline T-cell-mediated rejections. There were 54 rejection episodes in P0, 21 rejection episodes in P1, eight in P2, and 29 in P3. The clinical characteristics, including baseline characteristics and transplantation-related characteristics, are summarized in **Table 1**.

**Table 1:** Clinical characteristics of kidney transplant recipients.

Clinical characteristics	*N* = 192	% or ± SD
Baseline characteristics		
Age, mean	48.4	± 13.7
Male	122	63.5
Body mass index, mean	24.3	± 11.3
Underlying disease		
Diabetes mellitus	51	26.6
Pulmonary disease	9	4.7
Liver disease	7	3.6
Previous transplantation	3	1.6
ESRD cause		
Unknown	48	25
Hypertension	10	5.2
Diabetes	39	20.3
IgA nephropathy	37	19.3
Polycystic disease	11	5.7
Glomerulonephritis	43	22.4
Other	3	1.6
Dialysis		
Preemptive transplantation	38	19.8
Hemodialysis	125	65.1
Peritoneal dialysis	29	15.1
Transplant type		
cLDKT	104	54.2
iLDKT	53	27.6
DDKT	35	18.2
Mean admission days for operation	17.81	± 7.5
Sensitized patients		
PRA (>50%)	37	19.3
Preformed DSA	24	12.5
Desensitization total patients	50	26
Rituximab	48	
PPH + IVIG	32	
Induction with anti-thymoglobulin	25	13
Infection prophylaxis		
CMV prophylaxis	35	18.2
PCP prophylaxis	17	8.9
Rejection		
Experienced at least one rejection event	84	43.8
Rejection event	112	
Steroid pulse	97	
PPH	8	
IVIG	6	
Other treatments^a^	7	

ESRD, end-stage renal disease; cLDKT, compatible living donor kidney transplantation; iLDKT, incompatible living donor kidney transplantation; DDKT, deceased donor kidney transplantation; PRA, panel-reactive antibody; DSA, donor-specific antibody; CMV, cytomegalovirus; PCP, Pneumocystis jirovecii; PPH, plasmapheresis; IVIG, intravenous immunoglobulin.

a Other treatments include treatment with bortezomib, rituximab, or tocilizumab.

### 3.2 Serial changes in immunoglobulin and complement levels

**Figure 1:**
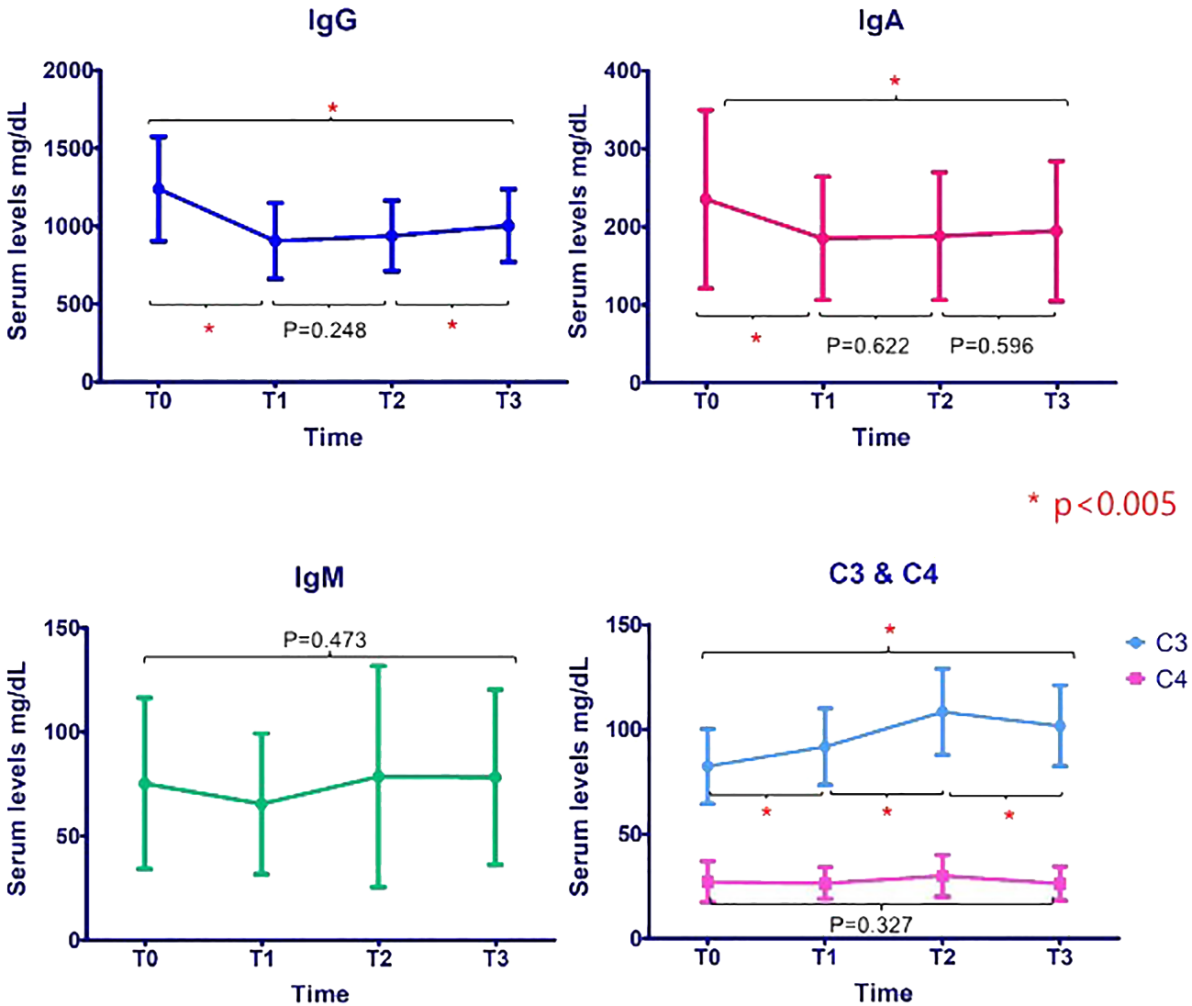
Changes in mean ( ± SD) levels of immunoglobulin and complement levels. *p<0.005.

The serum IgG, IgA, and C3 levels showed statistically significant changes over time with a *p*-value of less than 0.005. The mean values of serum IgG and IgA significantly decreased in P0 (T0 IgG 1,238.57 ± 336.05 mg/dL to T1 IgG 904.8 ± 243.8 mg/dL and T0 IgA 235.22 ± 114.38 mg/dL to T1 IgA 185.2 ± 78.8 mg/dL). The mean C3 levels significantly increased in P0 and P1 and then decreased in P2 (T0 = 82.3 ± 18.0 mg/dL, T1 = 91.6 ± 18.2 mg/dL, T2 = 108.3 ± 20.6 mg/dL, and T3 = 101.5 ± 19.3mg/dL). The mean levels of IgM and C4 did not change significantly (*p* = 0.473 and *p* = 0.327). The changes in the mean levels of immunoglobulin and complement levels are shown in **Figure 1**.

### 3.3 Prevalence of HGG and HCC

A total of 54 patients had HGG during the first year after transplantation. Among the 54 patients, eight (14.8%) had HGG at baseline, and half of these patients had persistent HGG throughout all four periods of detection. Excluding baseline HGG patients, a total of 46 (24.9%) of 192 patients had newly developed HGG in the post-transplant period (T1, *n* = 22; T2, *n* = 17; and T3, *n* = 7). HypoC3 was found in 118 patients, and among these patients, 62 also had hypoC4. A total of 98 (83.0%) of 118 patients had hypoC3 at T0, and only six of them had persistent hypoC3 throughout all four periods. Excluding baseline hypoC3 patients, only 20 (16.9%) patients had newly developed hypoC3 in the post-transplant period (T1, *n* = 18; T2, *n* = 2; and T3, *n* = 0). HypoC4 was found in 74 patients during the study period, and 37 (50.0%) of them had hypoC4 at T0. Excluding baseline hypoC4 patients, 37 (50.0%) patients had newly developed hypoC4 in the post-transplant period (T1, *n* = 21; T2, *n* = 6; and T3, *n* = 10). The total number of patients with both HGG and HCC was 15 (T0, *n* = 4; T1, *n* = 11, T2, *n* = 2; and T3, *n* = 2) and were too few for any further analysis.

### 3.4 Prevalence of infection

The total number of infectious episodes that occurred was 158 in 127 patients. **Table 2** shows the summary of types of infection by pathogen and clinical syndromes. Out of these cases, 36 (22.8%) were severe infections that required hospitalization, and 34 (21.5%) were graft-related viral infections. The primary pathogen for severe infection was bacterial in 75% (*n* = 27/36). In graft-related viral infection, the incidence of each viral infection was CMV 73.5% (*n* = 25/34), BKV 23.5% (*n* = 8/34), and EBV 2.9% (*n* = 1/34). Severe infection requiring hospitalization occurred most frequently in the P1 period (*n* = 19, 52.8%) and decreased with time (P2, *n* = 14 and P3, *n* = 3). Graft-related viral infection occurred most frequently in the P1 period (*n* = 19, 55.9%) and decreased with time (P2, *n* = 9 and P3, *n* = 6).

**Table 2:** Infectious episodes that occurred post-transplantation.

Posttransplant infection	Total *N* = 158 (%)
Infection by organism	
Virus	
CMV	25 (15.8)
BKV	8 (5.1)
VZV	8 (5.1)
Influenza virus	3 (1.9)
EBV	1 (0.6)
HSV-1	1 (0.6)
Bacteria	
*Escherichia coli*	12 (7.6)
*Klebsiella* spp.	7 (4.4)
*Mycobacterium tuberculosis* complex	3 (1.9)
*S*. *auerus*	3 (1.9)
Other nonfermenting gram-negative bacilli	3 (1.9)
Other Enterobacteriaceae	2 (1.3)
*S*. *pneumoniae*	1 (0.6)
*Enterococcus* spp.	1 (0.6)
*Pseudomonas* spp.	1 (0.6)
Fungi	
PCP	9 (5.7)
*Cryptococcus*	1 (0.6)
*Aspergillus fumigatus*	1 (0.6)
Infection by clinical syndrome	
Pneumonia and lower respiratory tract infection	20 (12.7)
Skin and soft tissue infection	13 (8.2)
Upper respiratory infection	6 (3.8)
Lower urinary tract infection	6 (3.8)
Acute pyelonephritis	6 (3.8)
Bloodstream infection	6 (3.8)
Intraabdominal infection	6 (3.8)
CMV viral syndrome	5 (3.2)
Other (nonspecific AGE, gastritis, and colitis)	5 (3.2)
Severe infection and graft-related viral infection	
Hospitalization	36 (22.8)
Graft-related viral infection	34 (21.5)

CMV, cytomegalovirus; BKV, BK virus; VZV, varicella zoster virus; EBV, Epstein–Barr virus; HSV, herpes simplex virus; PCP, pneumocystitis pneumonia.

### 3.5 Association between HGG and infection

**Figure 2:**
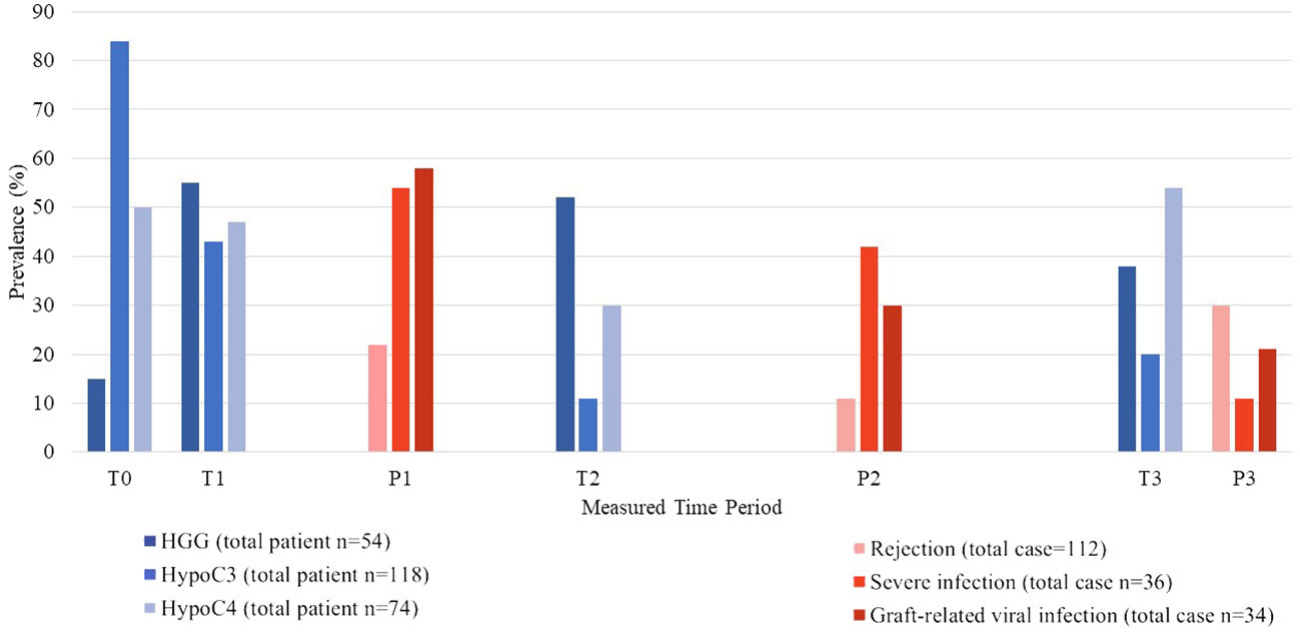
Prevalence of hypogammaglobulinemia and hypocomplementemia along with occurrence of infection and rejection episodes at each time period.

**Figure 2** shows the prevalence of HGG and HCC along with infection and rejection events at each period post-transplantation. In both univariate and multivariate analysis for factors associated with HGG, immune-modulating factors such as rejection treatment with IVIG and baseline HGG were significantly associated with HGG (**Table 3**). The single most associated factor related to HGG was presence of baseline HGG (OR 23.16, 95% CI: 7.508–71.461).

**Table 3:** Logistic regression of factors associated with hypogammaglobulinemia.

Associated factors	Univariate analysis			Multivariate analysis		
	OR	95% CI	*P*-value	OR	95% CI	*P*-value
Baseline factors						
Age	0.991	0.969–1.014	0.449			
BMI	0.942	0.877–1.013	0.108			
Dialysis state	2.257	0.974–5.234	0.058			
iLDKT	2.212	1.081–4.525	0.030			
Underlying disease						
DM	1.432	0.768–2.67	0.258			
Pulmonary disease	0.289	0.030–1.363	0.125			
Liver disease	2.141	0.656–6.746	0.201			
Previous transplantation	1.702	0.284–7.724	0.525			
Desensitization	1.228	0.599–2.518	0.575			
Induction with ATG	1.505	0.696–3.255	0.298			
Postoperative factors						
Rejection event	1.865	0.981–3.542	0.057			
Rejection treatment						
Steroid pulse	1.58	0.841–2.969	0.155			
PPH	6.882	3.151–15.034	<0.001			
IVIG	7.792	2.883–21.06	<0.001	5.702	1.783–18.235	0.003
Others^a^	5.815	3.225–10.483	<0.001			
Viral serology and prophylaxis						
CMV prophylaxis	1.962	0.989–3.89	0.054			
CMV D+/R-	1.389	0.416–13.256	0.775			
Immune markers						
Baseline HGG	21.333	9.763–66.520	<0.001	23.162	7.508–71.461	<0.001
HypoC3	1.485	0.705–3.126	0.298			
HypoC4	0.708	0.354–1.416	0.329			

BMI, body mass index; DM, diabetes mellitus; iLDKT, incompatible living donor kidney transplantation; D, donor; R, recipient; ATG, anti-thymoglobulin; PPH, plasmapheresis; IVIG, intravenous immunoglobulin; HGG, hypogammaglobulinemia; HypoC3, C3 hypocomplementemia; HypoC4, C4 hypocomplementemia.

a Other treatments include treatment with bortezomib, rituximab, or tocilizumab.

In the time-dependent Cox model analysis, the unadjusted analysis showed no significant association between HGG and infection. However, in the adjusted model with stabilized weights related to immunosuppression, HGG was significantly associated with infection. If HGG was present, the hazard ratio (HR) for hospitalization due to infection and graft-related viral infection was 1.895 (95% CI: 1.871–1.920; *p*< 0.001) and 1.152 (95% CI: 1.144–1.160; *p*< 0.001), respectively (**Table 4**).

**Table 4:** Time-dependent Cox proportional hazards model for hypogammaglobulinemia and its association with infection.

	HR	95% CI	*P*-value
Unadjusted analysis			
Hospitalization for infection	0.965	0.373–2.497	0.942
Graft related viral infection	1.465	0.667–3.217	0.341
CMV infection	1.760	0.742–4.175	0.1995
Adjusted analysis^a^			
Hospitalization for infection	1.895	1.871–1.920	<0.001
Graft related viral infection	1.152	1.144–1.160	<0.001
CMV infection	1.183	1.175–1.191	<0.001

CMV, cytomegalovirus.

a Adjusted analysis utilized stabilized weights that accounted for immune modulatory covariates which were rejection event occurrence, rejection treatment with intravenous steroid, rejection treatment with plasmapheresis and immunoglobulin, rejection treatment with anti-thymoglobulin, rejection treatment with other antibodies, preoperative desensitization including plasmapheresis, immunoglobulin usage and antibody treatment, and induction with anti-thymoglobulin.

## 4 Discussion

The serial assessment of immunoglobulins in kidney transplant recipients revealed significant temporal fluctuations, with the most pronounced decrease occurring at T1. The subsequent period correlated with peak incidences of hypogammaglobulinemia (HGG) and hypocomplementemia (HCC) along with severe infection and graft-related viral infections. Using a time-dependent Cox model with adjustments for confounders using stabilized weights from inverse probability treatment weighting (IPTW) delineated the specific impact of HGG on infection risk which was distinct from the effects of other immunomodulatory interventions.

Notably, in the risk factor analysis for post-transplant HGG, the single most associated factor related to HGG was the presence of baseline HGG. Rejection treatment with replacement of IVIG was another significant risk factor in the multivariate analysis. This association, however, likely reflects the overall heightened level of immunosuppression in patients receiving a more aggressive treatment for rejection, and further studies are warranted to elucidate the direct effects of IVIG replacement therapy on HGG. Additionally, other immune-modulating factors such as donor incompatibility and rejection treatment with plasmapheresis and other antibodies were also significant risk factors in univariate analysis. This indicates that the immunomodulatory interventions associated with high-risk transplants could contribute to altered immunoglobulin levels. Profound immunosuppression treatments lead to prolonged B-cell and T-cell depletion, and multiple studies have demonstrated an increased risk for graft-related viral infections (8, 18). Furthermore, serum immunoglobulin levels reflect the overall immunosuppressive status of the transplant recipient (10, 19). In the prospective multicenter study by Sarmiento et al., not only was there a decrease in serum immunoglobulin levels but also decreases in other pathogen-specific antibodies such as CMV antibody, and pneumococcal polysaccharide antibodies were also seen, and this overall secondary antibody deficiency resulted in increased infection (20). Therefore, attributing infection risk solely to HGG is an oversimplification. There exists a complex causal relationship between HGG, infection, and immune modulation.

By employing marginal structural Cox proportional hazards models with stabilized weights derived from inverse probability treatment weighting (IPTW), our research strived to clarify this complex causal relationship. The inclusion of both time-independent factors like preoperative desensitization and induction therapy and time-dependent factors such as rejection events and treatment allowed us to offer a more accurate estimation of the association between HGG and infection. The stabilized weights take into account the probabilities of having HGG and at each time point, given an individual’s covariate history. By accounting for these variables, our findings indicate a significant association between HGG and infection, suggesting that previous inconsistencies may be attributed to inadequate adjustment for such immune-modulating factors.

While this study identified HGG as a significant risk factor for infection in kidney transplant recipients, the role of IVIG replacement in mitigating this risk remains controversial. A small number of retrospective studies on the use of IVIG treatment in heart and lung transplant recipients with documented HGG have shown a reduced re-infection rate or mortality among those treated (21, 22). IVIG replacement has demonstrated potential in preventing specific viral infections in a recent publication on the clinical trial on using IVIG vs. immunosuppressive reduction to treat BK viremia post-kidney transplantation. Patients treated with IVIG showed markedly lower BK viremia and BK nephropathy at 12 months post-transplant (23). In contrast, a randomized trial of IVIG treatment in patients with HGG after lung transplantation by Lederer did not show any effect on bacterial infection or any other infections and only significantly increased IgG levels (24). As stated by Bourassa‐Blanchette et al., the efficacy seems inconsistent across different transplant types and infection contexts, and many of the existing studies are constrained by small sample sizes (25). There is a need for larger, well-designed prospective studies to ascertain the true impact of IVIG therapy on infection rates and outcomes in transplant recipients with HGG.

There are several notable limitations of this study. Unlike other studies, non-pathogen-specific, composite end points were used in this study. The retrospective aspect limited the number of infectious episodes that could be analyzed alongside serial immunoglobulin levels. Consequently, we defined outcomes that have largest clinical significance. Infections requiring hospitalization admission are cases in which the patients are unstable, require long-term intravenous treatment, and have poor outcomes than patients that can be treated in outpatient clinics. Additionally, graft-related viral infections involve viruses with lifelong latencies and exert indirect detrimental impacts on both patient and graft outcomes (10). Monitoring and controlling immunosuppressive agents meticulously are necessary to prevent the reactivation of these viruses (10, 14). Nevertheless, 71% of severe infection cases were of bacterial origin, and 67.6% of graft-related viral infection cases were CMV-infected cases. Similarly, our study showed associations of immunoglobulin with bacterial infection and CMV, consistent with previous studies (3, 7, 26). Another limitation of the retrospective aspect of this study was the variability of FK C0 levels, making it difficult to prove its relationship to infection, yet previous studies have reported that maintenance immunosuppressants are not risk factors for HGG as they directly alter T-cell function (2, 5). Other key immune-modulating therapies such as desensitization and rejection treatments were adjusted using an advanced statistical approach to modify time-varying covariates. Due to the limitation in case number, certain immune modulating factors such as type of rejection and different desensitization protocols and treatments were clustered into single large categories. A larger-scale data is warranted to identify the degree and effect of secondary immune deficiency on these high-risk patients.

Immune monitoring strategies through immunoglobulins or complement levels can offer a more dynamic insight into the net state of immunosuppression of transplant recipients. Furthermore, the presence of HGG in the post-transplant period was identified as a risk factor for clinically significant infections. Therefore, serum immunoglobulins can serve as a feasible biomarker for infection.

## Data availability statement

The raw data supporting the conclusions of this article will be made available by the authors, without undue reservation.

## Ethics statement

The studies involving humans were approved by Institutional Review Board at Seoul National University Hospital. The studies were conducted in accordance with the local legislation and institutional requirements. The human samples used in this study were acquired from a by-product of routine care or industry. Written informed consent for participation was not required from the participants or the participants’ legal guardians/next of kin in accordance with the national legislation and institutional requirements.

## Author contributions

EJ: Conceptualization, Data curation, Formal analysis, Investigation, Methodology, Visualization, Writing – original draft, Writing – review & editing. SM: Conceptualization, Funding acquisition, Investigation, Project administration, Supervision, Writing – review & editing. AJ: Formal analysis, Validation, Writing – review & editing. AH: Data curation, Resources, Visualization, Writing – review & editing. JH: Conceptualization, Resources, Supervision, Writing – review & editing. ES: Data curation, Investigation, Resources, Writing – review & editing. HL: Resources, Writing – review & editing. YK: Investigation, Resources, Writing – review & editing.
